# Light-Driven Tetra- and Octa-β-substituted Cationic Zinc(II) Phthalocyanines for Eradicating *Fusarium oxysporum* Conidia

**DOI:** 10.3390/ijms242316980

**Published:** 2023-11-30

**Authors:** Leandro M. O. Lourenço, Ângela Cunha, Isabel N. Sierra-Garcia

**Affiliations:** 1 LAQV-REQUIMTE, Department of Chemistry, University of Aveiro, 3810-193 Aveiro, Portugal; 2 CESAM, Department of Biology, University of Aveiro, 3810-193 Aveiro, Portugal; acunha@ua.pt (Â.C.); inatalia.sierra@ua.pt (I.N.S.-G.)

**Keywords:** photodynamic inactivation (PDI), *Fusarium oxysporum* conidia, quaternized phthalocyanines, reactive oxygen species (ROS), singlet oxygen

## Abstract

Photodynamic inactivation (PDI) is an emerging therapeutic approach that can effectively inactivate diverse microbial forms, including vegetative forms and spores, while preserving host tissues and avoiding the development of resistance to the photosensitization procedure. This study evaluates the antifungal and sporicidal photodynamic activity of two water-soluble amphiphilic tetra- and octa-β-substituted zinc(II) phthalocyanine (ZnPc) dyes with dimethylaminopyridinium groups at the periphery (ZnPcs **1**, **2**) and their quaternized derivatives (ZnPcs **1a**, **2a**). Tetra(**1**, **1a**)- and octa(**2**, **2a**)-β-substituted zinc(II) phthalocyanines were prepared and assessed as photosensitizers (PSs) for their effects on *Fusarium oxysporum* conidia. Antimicrobial photoinactivation experiments were performed with each PS at 0.1, 1, 10, and 20 µM under white light irradiation at an irradiance of 135 mW·cm^–2^, for 60 min (light dose of 486 J·cm^−2^). High PDI efficiency was observed for PSs **1a**, **2**, and **2a** (10 µM), corresponding to inactivation until the method’s detection limit. PS **1** (20 µM) also achieved a considerable reduction of >5 log_10_ in the concentration of viable conidia. The quaternized PSs (**1a**, **2a**) showed better PDI performance than the non-quaternized ones (**1**, **2**), even at the low concentration of 1 µM, and a light dose of 486 J·cm^−2^. These cationic phthalocyanines are potent photodynamic drugs for antifungal applications due to their ability to effectively inactivate resistant forms, like conidia, with low concentrations and reasonable energy doses.

## 1. Introduction

*Fusarium* is a genus of filamentous fungi that are commonly found in soil and plants. Within this genus, some species cause diseases in plants, animals, and humans [1,2,3,4,5]. *Fusarium*-related diseases have a substantial economic impact on a wide range of crops, including fruits, vegetables, cereals, and cellulose production [3,6,7]. In humans, *Fusarium oxysporum* is an opportunistic pathogen, responsible for conditions such as keratitis, onychomycosis, and invasive infections in both immunocompromised and immunocompetent individuals [4,8,9].

The asexual reproductive cycle of *Fusarium oxysporum* involves the formation of chlamydospores, macroconidia, and microconidia, which facilitate effective dispersion in the environment [10]. Plant infections primarily take place via the roots [11]. Conidia are dispersed by wind or rain and germinate in the rhizosphere soil. The advancing hyphae infiltrate root tissues and initiate the infection process [10]. Some strains of *F. oxysporum* are capable of producing mycotoxins, including fumonisins, enniatins, and beauvericin [12]. These mycotoxins can have harmful effects on both plants and human and animal health if contaminated plant material is ingested [13]. However, not all isolates of this fungus can produce mycotoxins. The mycotoxin profile varies among strains and may determine host specificity [14]. Typically, conventional fungicides are designed to address conidial germination and the early stages of development [15].

In managing *Fusarium* diseases in crops, the use of plant varieties that possess inherent resistance is the primary tool. This is due to the growing resistance of *Fusarium* to common chemical biocides. However, the level of resistance may differ based on the specific environmental conditions in which these plants are cultivated [16,17,18]. In certain regions, even when resistant plant varieties are employed, the threat of *Fusarium* spp. colonization can intensify under elevated temperatures [19]. Other commonly used strategies include pre-treating the seeds and seedlings with fungicides before planting, and applying fungicides throughout the crop development process [19,20]. However, these strategies are becoming less reliable due to the emergence of more tolerant fungal strains. Furthermore, the extensive use of current fungicides is viewed as a potential risk to both human health and the environment [6,7,9]. To mitigate the possibility of fungal contamination in postharvest crops, it is customary to subject them to treatments with chlorine or organic acids. However, these treatments can also have adverse effects on the environment [11,21].

The rising resistance of fungal pathogens to commonly used fungicides, coupled with the gradual prohibition of popular pesticides in the European Union, presents a significant constraint to chemical controls of fungal diseases in crops [22,23,24]. Biocontrol agents show promise for managing fungal plant diseases sustainably. However, their effectiveness is highly dependent on environmental conditions [25]. Consequently, there is a growing interest in the development of more effective technologies for managing pathogenic fungi. Photodynamic inactivation (PDI) has gained increasing attention as an alternative method for inactivating microorganisms within environmental contexts [26,27]. It has been proven to be successful in eradicating bacteria and fungi in animal hosts and their surrounding environments [28]. PDI relies on three harmless components: a photosensitizer (PS), visible light, and oxygen (^3^O_2_). The combination of these elements generates reactive oxygen species (ROS), such as singlet oxygen (^1^O_2_) and free radicals. These ROS cause lethal oxidative damage to microbial targets, including lipids, proteins, and nucleic acids, leading to the death of the target cells without significantly harming the host cells [29].

The utilization of PDI to combat plant pathogens represents a recent advancement in this technique, offering a promising alternative to toxic agrochemicals. PDI has shown to be effective against bacterial diseases, such as kiwifruit cancer [30] and citrus cancer [31], as well as phytopathogenic fungi such as *Lasiodiplodia theobromae* (the causative agent of vine trunk disease), *Botrytis cinerea* (associated with plant necrosis), and *Colletotrichum* sp. (responsible for anthracnose in various fruit trees) [32,33,34]. Fungal spores play a critical role in spreading fungal diseases, with fungal conidia being a significant target for photosensitization [35].

Investigating the structure–activity relationship plays is essential for developing potent photosensitizers (PSs) capable of inflicting lethal damage on plant pathogens within brief exposure to irradiation without harming host plant tissues. Porphyrin (Por) [33], chlorin (Chl) [36,37], and phthalocyanine (Pc) [38,39] dyes have been extensively employed in the PDI method. Pcs exhibit distinctive UV-visible spectra, typically characterized by a Soret band with a maximum wavelength at 350 nm and intense Q bands within the red/near-infrared range (600–800 nm) [38,39,40]. The physicochemical properties and biological activities of Pcs are significantly influenced by their structural features and specific functionalities. Modifying the Pc macrocycle by introducing different quaternized peripheral groups or incorporating metallic ions (e.g., Zn(II)), which can enhance the triplet excited state and singlet oxygen quantum yield, are reliable strategies for fine-tuning the physicochemical attributes of Pcs for improving efficacy against microbial targets [29,40].

The interaction of a PS with fungal spores is influenced by the overall hydrophobicity of the spore coating and the presence of charged groups within the PS structure [36,41]. Consequently, a PS can either remain adsorbed to the outer layers of spores (or vegetative hyphae) or penetrate the intracellular compartment, which expands the range of physiological and biochemical targets [33,42]. Conidia are a type of asexual spore produced by fungi. They are typically formed as a protective structure offering higher resistance to oxidative stress compared to prokaryotic cells [43], but also providing a broader array of subcellular targets for photosensitization [44]. Therefore, a photosensitizer with multi-target capability can overcome the intrinsic resistance of fungal spores to photosensitization [45].

This study investigates the antifungal photodynamic efficacy of four cationic zinc(II) Pc derivatives [46] against *Fusarium oxysporum* conidia and determines the relationship between the number of cationic peripheral substituents and the amount of positive charges on the tetra(**1**, **1a**)- and octa(**2**, **2a**)-substituted zinc(II) phthalocyanines.

## 2. Results

### 2.1. Synthesis and Photophysical Analysis of Phthalocyanine Dyes

**Figure 1:**
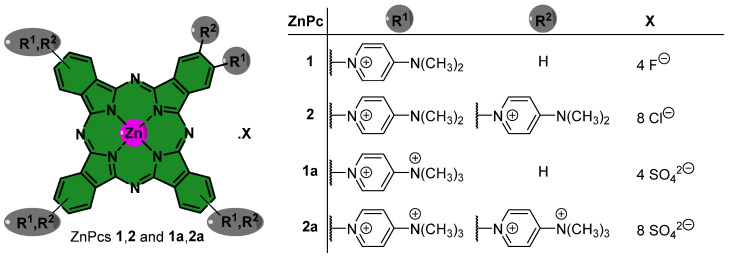
Structures of cationic tetra- and octa-substituted ZnPcs **1**, **1a** and **2**, **2a**, respectively.

**Figure 2:**
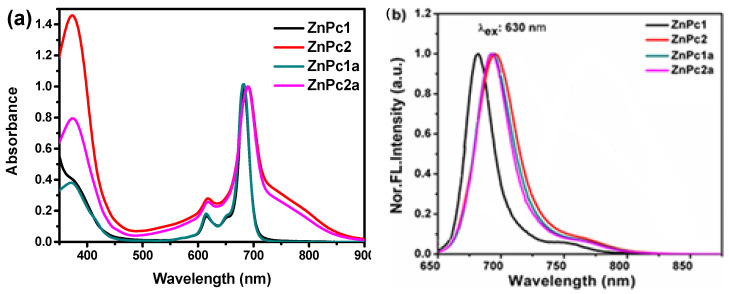
Normalized electronic spectra of (**a**) absorption and (**b**) emission spectra for compounds **1**, **1a** and **2**, **2a** in DMSO at 298 K (λ_exc_ = 630 nm).

Tetra(**1**, **1a**)- and octa(**2**, **2a**)-β-substituted zinc(II) phthalocyanines (Figure 1) were successfully synthesized and characterized using NMR techniques, following previously established protocols [46]. Absorption and emission spectra for **1**, **1a** and **2**, **2a** were acquired in dimethyl sulfoxide (DMSO) at low concentrations (10^−6^ M). The absorption spectra (Figure 2a) displayed the characteristic features of zinc(II) phthalocyanines, with a prominent Soret band spanning the 350–450 nm range and robust Q bands within the 600–800 nm range. When excited at different wavelengths, both ZnPcs exhibited emission bands with maxima between 684 and 696 nm (Figure 2b). Notably, the fluorescence quantum yields (Φ_F_) of PSs **1**, **1a** and **2**, **2a** in DMSO were between 0.11 and 0.24 [46], compared to **ZnPc** as standard reference (Φ_F_ = 0.20 in DMSO) [47].

Considering the potential utilization of PSs **1**, **1a** and **2**, **2a** as agents targeting *Fusarium oxysporum* conidia, it was imperative to evaluate their capability to generate singlet oxygen species. A previous assessment of the ^1^O_2_ production capacity of these Pc derivatives [46], determined through the indirect method of measuring the absorption decay of 1,3-Diphenylisobenzofuran (DPBF), revealed that quaternized ZnPcs **1a** and **2a** had higher photosensitizing efficacy (65 and 89%, respectively) than non-quaternized ZnPcs **1** and **2** (35 and 40%, respectively).

### 2.2. Photodynamic Inactivation of Fusarium oxysporum Conidia

**Figure 3:**
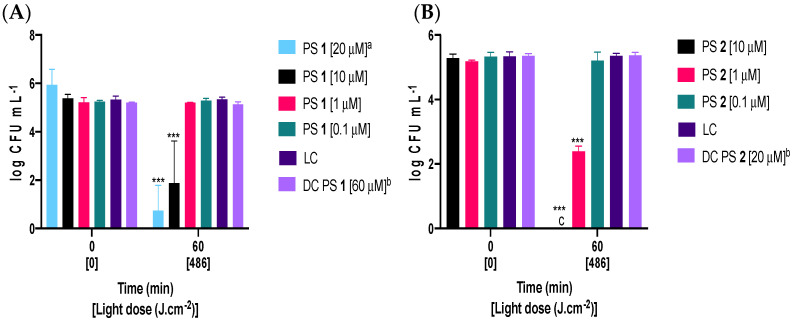
Variation in the concentration of viable conidia of *Fusarium oxysporum* after 60 min of irradiation with artificial white light, at a fluence rate of 135 mW·cm^−2^ (light dose of 486 J·cm^−2^) in the presence of (**A**) 0.1, 1, 10, and 20 μM of PS **1** or (**B**) 0.1, 1, and 10 μM of PS **2**. LC, light control; DC, dark control, respectively. Values correspond to the average of three independent experiments with replicates. Error bars represent the standard deviation. ^a^ assay performed in duplicate; ^b^ one assay with three analytic replicates; ^c^ no colonies observed; *** significance < 0.0001.

**Figure 4:**
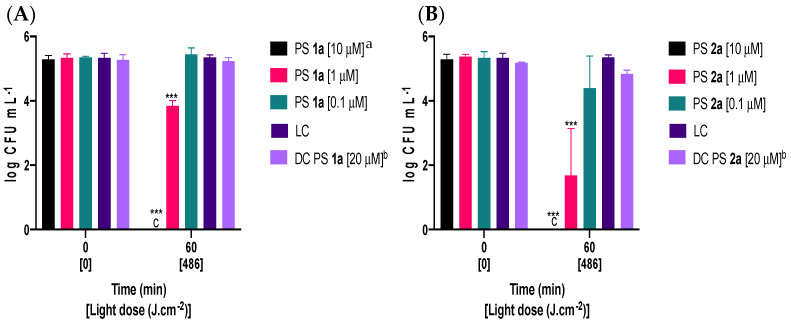
Variation in the concentration of viable conidia of *Fusarium oxysporum* after 60 min of irradiation with artificial white light, at a fluence rate of 135 mW·cm^−2^ (light dose of 486 J·cm^−2^) in the presence of 0.1, 1, and 10 μM for PSs (**A**) **1a** or (**B**) **2a**. LC, light control; DC, dark control. Values correspond to the average of three independent experiments with replicates. Error bars represent the standard deviation. ^a^ assay performed in duplicate; ^b^ one assay with three analytic replicates; ^c^ no colonies observed; *** significance < 0.0001.

Figure 3 and Figure 4 display the logarithmic decrease in the number of viable *Fusarium oxysporum* conidia after 60 min of exposure to artificial white light with a fluence rate of 135 mW·cm^−2^ (light dose of 486 J·cm^−2^). Experiments were conducted in the presence of tetra-substituted PSs **1**, **1a** and octa-substituted PSs **2**, **2a** at concentrations of 0.1, 1, 10, and 20 μM. It is noteworthy that all PSs were stable and photostable during the experiments, and all were soluble in aqueous media (verified by the Beer-Lambert law) [46]. After 60 min of irradiation, the tetra-substituted PS **1** with a concentration of 20 μM led to a significant reduction (~5 log_10_) in the concentration of viable conidia. When the concentration was halved to 10 μM, it still caused a 3 log_10_ inactivation. Only octa-substituted PS **2**, at a concentration of 10 μM, achieved complete inactivation of conidia, down to the detection limit. At the lowest tested concentrations of 1 and 0.1 µM, only octa-substituted PS **2** (1 µM) exhibited a significant effect on conidia viability, resulting in approximately 3 log_10_ reduction. None of the PSs caused lethal damage at the highest tested concentration in the absence of light (dark controls: DC PS **1** and DC PS **2**, Figure 3A,B).

To determine the effectiveness of quaternized tetra-substituted PS **1a** and octa-substituted PS **2a** in inactivating *Fusarium oxysporum*, the concentration of viable conidia was evaluated before and after 60 min of irradiation with white light (Figure 4). Both PSs **1a** and **2a**, at a concentration of 10 μM, were able to completely inactivate conidia, reaching the detection limit of the method. Within 60 min of exposure to light, PS **2a** was found to be more effective than PS **1a** at a concentration of 1 μM, causing a reduction of approximately 3 log_10_ in comparison to only 1.5 log_10_ in conidia viability, respectively. However, at the lowest tested concentration (0.1 μM), PS **2a** did not show any significant inactivation, with only a slight decrease of approximately 1 log_10_ in conidia viability. At the highest tested concentrations of PSs **1a** and **2a**, neither compound induced lethal damage in the absence of light, as shown in dark control conditions (DC PS **1a** and DC PS **2a**, Figure 4).

## 3. Discussion

The rise in fungal resistance to traditional antifungal treatments, specifically in conidia which are responsible for pathogen spread [48], has resulted in a significant amount of research being conducted to find new, effective, and environmentally friendly methods to control them. One such method is PDI. The efficiency of photosensitization heavily relies on the structure of PS molecules [49,50,51,52]. In particular, the presence of positive charges is crucial to enhancing the water solubility of PSs and achieving effective photosensitization of fungal targets [49,53]. In this regard, cationic tetra- and octa-substituted ZnPcs **1**, **1a** and **2**, **2a** (shown in Figure 1), were synthesized [46] and tested against conidia of *Fusarium oxysporum*, a model fungal pathogen. The effectiveness of PDI was quantified by assessing the logarithmic reduction in viable conidia for different PS concentrations (Figure 3 and Figure 4) under irradiation (60 min, 135 mW·cm^−2^, 486 J·cm^−2^).

With irradiation in the absence of PS, light alone did not induce a significant inactivation of conidia, and reversely no inactivation was observed in the absence of light and the presence of the highest PS concentration (LC, DC PSs **1**, **1a** and DC PSs **2**, **2a**, Figure 3 and Figure 4). Light does not affect the germination and growth of *Fusarium* conidia, although the light regime is known to modulate conidiation and tolerance of conidia to UV [54] and to induce the expression of light-protective metabolites, like carotenoids [55].

After 60 min under light exposure (light dose of 486 J·cm^−2^), the lowest tested concentration (0.1 µM) failed to cause any significant conidia inactivation. In order to attain lethal damage, higher concentrations of each PS (1, 10, or 20 μM) were assessed.

With a concentration of 1 µM of PS, and similar irradiation conditions (light dose of 486 J·cm^−2^), a significant inactivation could be observed with all tested PSs, except for PS **1**, which represents the first evidence of the lower efficiency of this PS, in comparison with the other molecules. An estimate of the minimum inhibitory concentration (MIC) of thiopyridinium or ammonium phthalocyanines against conidia, pointed to values of 5 to 60 µM [38,39]. Therefore, assays conducted with a concentration of 1 µM may be interpreted as representing sub-lethal conditions, and allow the comparative assessment of the different PSs. A greater reduction in the concentration of viable conidia (~3.5 log_10_) was observed with PS **2a** (1 µM, Figure 4B), ranking this PS as the most effective against *Fusarium oxysporum* conidia.

A further 10-fold increase in concentration of PS (10 µM), led to the complete inactivation of conidia with PSs **2**, **1a**, and **2a** as illustrated in Figure 3B and Figure 4A,B, respectively. With 10 μM of PS **1,** the inactivation corresponded to a ~4 log_10_ reduction in the concentration of viable conidia (Figure 3A). The moderate performance of PS **1** (10 µM, Figure 3A) in comparison to the high performance of PS **1a** (10 µM, Figure 4A) may be interpreted as an indication that quaternization improves photosensitization capacity against conidia. An even higher concentration of PS (20 µM) was tested only for PS **1**. Although a significant reduction in the concentration of viable conidia was observed (~5 log_10_), complete inactivation was still not attained. In the case of these PSs (**1**, **1a** and **2**, **2a**), an even higher concentration may be required, since the increase in concentrations, with a light dose of 486 J·cm^−2^, confirmed that the cationic PSs efficiently inactivate *Fusarium oxysporum* conidia.

Comparing the obtained results with non-quaternized PSs **1**, **2** (Figure 3A,B) and quaternized PSs **1a**, **2a** (Figure 4A,B) at a sub-lethal concentration (1 μM) shows that quaternized PSs **1a** caused >1 log_10_ reductions, whereas non-quaternized Ps **2** caused a <2.5 log_10_ decrease in the concentration of viable conidia and PS **1** caused no reduction at all. An investigation of inactivation of a Gram-negative bacterial model, *Escherichia coli*, in both planktonic and biofilm forms, also confirmed that quaternized PSs **1a**, **2a** were more efficient than the non-quaternized ones when a concentration of 20 µM was used (PSs **1**, **2**) [46]. This fact is most probably associated with the difference in the ^1^O_2_ production (non-quaternized PSs **1**, **2** < quaternized PSs **1a**, **2a**) and positive double-charge of each substituent that maximizes the electrostatic interactions of the quaternized derivatives (ZnPcs **1a**, **2a**) with the fungal spores.

The effect of the increase in the number of charges on the photosensitization capacity can be inferred from the comparison of the obtained results with tetra-substituted and octa-substituted PSs. In the octa-substituted PS **2** (Figure 3B), despite being considered an antimicrobial agent, the introduction of more cationic substituents to the β-position seemed to increase the antifungal activity when compared with tetra-substituted PS **1** (Figure 3A), which was well-observed at 1 and 10 μM and could be correlated with the previously mentioned difference in the ^1^O_2_ production (ZnPc **1** < ZnPc **2**) and electrostatic interactions (four versus eight positive charges) with the target conidia.

Both PSs **1a** and **2a** led to the complete inactivation of conidia with a concentration of 10 μM (Figure 4A,B). In order to compare them in terms of inactivation efficiency, results obtained with lower concentrations need to be considered. At the lowest tested concentration (0.1 μM), PS **1a** did not show any effect (Figure 4A), and PS **2a** exhibited a slight inactivation (Figure 4B), displaying only a marginal reduction of approximately ~1 log_10_ in conidia viability. This suggests that PS **2a** is slightly more effective than PS **1a**. This trend is confirmed by the obtained results with a higher, but still sub-lethal, concentration (1 µM), indicating a higher photosensitization capacity of PS **2a** (~3.5 log_10_ reduction) in comparison to PS **1a** (~1.5 log_10_ reduction). Overall, this suggests that the increased number of positive charges on PS **2a** possibly leads to an increased ^1^O_2_ production and stronger electrostatic interactions, which enhances the photoinactivation of conidia.

Regardless of differences in the photosensitization efficiency, which can be related to ^1^O_2_ generation capacity and the effect of the number of charges on solubility and affinity towards the spore material, all PSs have been shown to be effective against *Fusarium oxysporum* conidia at concentrations above 10 µM. Other studies of the photoinactivation of *Fusarium oxysporum* conidia with the use of ammonium phthalocyanines as photosensitizers needed higher concentrations of PS such as 40 and 60 μM at the same light doses [38]. The study found that quaternized derivatives were highly effective as photosensitizers and required only a concentration of 1 μM to significantly inactivate *Fusarium oxysporum* conidia. Although typically higher concentrations are required to photosensitize fungal structures, both non-quaternized PSs **1**, **2** and quaternized PSs **1a**, **2a** probably target a diverse range of components of conidia, leading to high antifungal activity. These results suggest that a wide-range, multi-organism phytosanitary strategy, based on cationic zinc(II) phthalocyanines, may be possible.

It is noteworthy that fungal spores and vegetative forms (hyphae) have varying levels of susceptibility to PDI. Hyphae may indeed be more resistant to PDI than spores, depending on the nature and affinity of the PS to the target structures [56]. In the case of *Fusarium*, conidia are infectious forms, and the macroconidia are responsible for spreading the infection over long distances [57]. Infection begins when spores germinate and penetrate plant tissues through the roots without symptoms [58]. Therefore, the inactivation of conidia is considered the most straightforward and long-lasting way to control fungal infections in crops [34,59,60]. In future research, it will be necessary to evaluate the affinity of cationic phthalocyanines for both conidia and mycelium and to determine the susceptibilities of these structures to photosensitization. This information can be used to improve the design of phytosanitary protocols.

## 4. Materials and Methods

### 4.1. Synthesis and Photophysical Characterization of the Photosensitizers

The molecular structures of the cationic PSs bearing cationic groups, designated as **1**, **1a** and **2**, **2a**, are presented in Figure 1. The synthesis **1**, **1a** and **2**, **2a** was conducted following previously established experimental procedures [46], using reagents of high purity, procured from Merck, Steinheim, Germany. Analytical thin-layer chromatography (TLC) was performed on pre-coated silica gel sheets with a thickness of 0.2 mm (Merck, Darmstadt, Germany). As per the literature, solvents were either used in their as-received state or subjected to distillation and dehydration procedures [61]. ^1^H and ^19^F NMR spectra were recorded on a Bruker Avance-300 spectrometer based in Wissembourg, France, operating at 300.13 and 282.38 MHz, with tetramethylsilane (TMS) serving as the internal reference. Absorption and steady-state fluorescence spectra were acquired using a Shimadzu UV-2501PC spectrophotometer (Shimadzu, Kyoto, Japan) and a Horiba Jobin-Yvon FluoroMax Plus spectrofluorometer (Horiba Ltd., Kisshoint, Japan), respectively. The absorption and emission spectra of **1**, **1a** and **2**, **2a** were measured in DMSO within quartz optical cells with dimensions of 1 × 1 cm, at a temperature of 298.15 K. Φ_F_ for **1**, **1a** and **2**, **2a** was determined in DMSO by comparing the area beneath the corrected emission spectra to that of **ZnPc**, which served as the standard (Φ_F_ = 0.20 in DMSO) [47].

### 4.2. Photosenstizer Stock Solutions

Stock solutions of the PSs at a concentration of 500 µM were prepared in either DMF for photophysical analyses or DMSO for photodynamic inactivation experiments. These solutions were stored in a light-protected environment and were pre-treated by ultrasonic sonication for 30 min before each assay.

### 4.3. Light Source

All photodynamic inactivation experiments were conducted by subjecting the samples and light controls to white light within the range of 400–800 nm. The light was delivered through a fiber-optic probe connected to a 150 W quartz/halogen lamp (model LC122, LumaCare™ MBG Technologies Inc., New Port Beach, CA, USA). The irradiance, measured at 135 mW·cm^–2^, was determined using a Coherent FieldMaxII-Top energy meter in conjunction with a Coherent PowerSensPS19Q energy sensor.

### 4.4. Preparation of Stock Suspensions of Fusarium oxysporum Conidia

*Fusarium oxysporum* was cultivated in potato dextrose agar (PDA, Merck, KGaA, Darmstadt, Germany) for 7 days at 25 °C, following a previously described procedure [36]. To ensure the absence of hyphae in the conidia suspensions, a microscopic examination was performed using a Leitz Laborlux K microscope from Ernst Leitz GmbH, Wetzlar, Germany. The concentration of viable conidia was determined by subjecting an aliquot to serial dilutions in PBS (pH 7.4), and spread-plating on Rose Bengal chloramphenicol agar (Merck, KGaA, Darmstadt, Germany). After a 2-day incubation at 25 °C, colonies were counted, and the concentration of conidia was expressed as colony-forming units per milliliter (CFU·mL^−1^) of the suspension.

The fungal strain was provided by the Fungi and Plant Biology Laboratory—FunPlantLab of the Department of Biology, University of Aveiro. The strain was isolated from pine trees in Portugal. The spore suspensions were prepared following the procedure previously described in the literature [36]. To extract the conidia from PDA (Merck) cultures, 5 mL of phosphate-buffered saline (PBS) was added to 10 cm plates. Then, the mycelium was gently scraped using a sterilized glass spreader. The suspension was passed through sterilized cotton gauze to filter out hyphae and other debris, and concentrated by centrifugation (4000× *g*). The absence of hyphal material and a negligible proportion of microconidia was verified under light microscopy. The concentration of conidia in the suspension was determined by colony counting after serial dilution and spread-plating on Rose Bengal chloramphenicol agar (VWR, Leuven, Belgium). After an incubation period of 2 days at 25 °C, the colonies were counted, and the concentration of conidia was expressed as CFU·mL^−1^. The spore suspensions were stored at −20 °C until the experiment and then diluted with sterile PBS to achieve a suitable volume, with a concentration of spores of approximately 10^5^ CFU·mL^−1^.

The microscopy inspection of the suspensions revealed that the presence of microconidia was negligible, as expected from the methods used to obtain conidia from stock cultures. Microconidia are smaller, unicellular, and often produced directly on the hyphae, and are more tightly attached to it compared to macroconidia. The latter are larger, multicellular (septated), and produced in specialized structures called macroconidiophores, which are separate from the mycelium. These structures aid in the long-distance dispersal of the loosely attached macroconidia [59,62]. The procedure of gently scraping the mycelium will more efficiently detach macroconidia, which can be easily washed with the buffer. Furthermore, the microconidia attached to the hyphae will be retained in the gauze upon filtration, and the low-speed centrifugation will further contribute to the selective precipitation of the large macroconidia.

### 4.5. Photodynamic Inactivation Assays

The photoinactivation assays were performed on PBS suspensions containing approximately 3 × 10^5^ CFU·mL^−1^ with a range of concentrations of 0.1, 1, 10, or 20 µM for PSs **1**, **1a** and **2**, **2a**. The tests were carried out in 24-well plates in a final volume of 1.5 mL of suspension. Conidia suspensions were preincubated with the PS, in the dark, for 30 min at room temperature, with magnetic gentle stirring. After the preincubation, light exposure was conducted for 1 h of constant irradiation. During irradiation, the suspension was kept under stirring on melting ice, to prevent heating. Aliquots of 100 μL were collected at the beginning (t = 0 min) and at the end of the irradiation (t = 60 min), serially diluted in PBS and spread-plated on Rose Bengal Chloramphenicol Agar, in triplicate, for the determination of the concentration of viable spores. Colonies were counted in the most convenient dilution after 48 h incubation at 25 °C. The average of the colonies in the replicates was used to estimate the concentration of viable conidia in the suspension expressed as CFU·mL^−1^. Two controls were included in each experiment: a light control (LC) submitted to the same irradiation conditions as the samples but without PS, and a dark control (DC) containing the highest PS concentration, but kept in the dark. Three independent assays were conducted for each PS. The inactivation efficiency was determined as the logarithmic (log_10_) reduction in the concentration of viable *Fusarium oxysporum* conidia during the corresponding irradiation period for each independent assay.

### 4.6. Statistical Assessment

The significance of inactivation (difference between the initial and final concentrations of viable conidia) was evaluated using a two-way univariate analysis of variance (ANOVA) model, followed by Tukey’s multiple-comparisons post hoc test. Significance was established at a threshold of *p* < 0.05.

## 5. Conclusions

The relations between structural features and the efficiency of photosensitization of *Fusarium oxysporum* conidia indicated that cationic PSs **1a**, **2**, and **2a** (10 µM) were more efficient (reduction in the concentration of viable conidia down to the detection limit of the method) than the PS **1** (20 µM, achieving a reduction of ~5 log_10_ in the conidia viability). The different photodynamic activity against fungi was related to the ability to generate ^1^O_2_ species (following the increasing order of **1** (35%) < **2** (40%) < **1a** (65%) < **2a** (89%)) and with the electrostatic interactions (attending to the number of charges and their charge position on the peripheral substituent on the Pc structure). In this study, doubling the number of charges in the same peripheral substituent (PSs **1**, **2** vs. PSs **1a**, **2a**) improved the photoinactivation process. The obtained findings provide a solid foundation for considering these positively charged PSs as promising candidates for novel phytosanitary agents, relying on the photodynamic management of fungal spores.

## Data Availability

Data is contained within the article.
